# Comparison of Immune Checkpoint Molecules *PD-1* and *PD-L1* in Paired Primary and Recurrent Glioma: Increasing Trend When Recurrence

**DOI:** 10.3390/brainsci12020266

**Published:** 2022-02-14

**Authors:** Wei Yu, Anwen Shao, Xiaoqiu Ren, Zexin Chen, Jinghong Xu, Qichun Wei

**Affiliations:** 1 Department of Radiation Oncology, Key Laboratory of Cancer Prevention and Intervention, Ministry of Education, Second Affiliated Hospital, Zhejiang University School of Medicine, Hangzhou 310009, China; wei-yu@zju.edu.cn (W.Y.); rxq@zju.edu.cn (X.R.); 2 Department of Neurosurgery, The Second Affiliated Hospital, Zhejiang University School of Medicine, Hangzhou 310009, China; 2316040@zju.edu.cn; 3 Center of Clinical Epidemiology and Biostatistics for Statistical Analysis, The Second Affiliated Hospital, Zhejiang University School of Medicine, Hangzhou 310009, China; chenzexin@zju.edu.cn; 4 Department of Pathology, The Second Affiliated Hospital, Zhejiang University School of Medicine, Hangzhou 310009, China; zydxjh@zju.edu.cn

**Keywords:** glioma, recurrence, programmed cell death 1 receptor, programmed death-ligand 1, therapeutics

## Abstract

**Purpose:** This study aims to investigate *PD-1/PD-L1* expression patterns in paired primary and recurrent gliomas. **Methods:** From January 2008 to December 2014, 42 patients who underwent surgical resections of primary and recurrent gliomas were retrospectively included. *PD-1/PD-L1* protein expression in tumors was evaluated through immunohistochemistry. **Results:** In primary gliomas, *PD-1* and *PD-L1* expression was evident in 9 (22.0%) and 14 (33.3%) patients. In the paired recurrent glioma, *PD-1* and *PD-L1* expression was evident in 25 (61.0%) and 31 (74.0%) lesions. Both *PD-1* and *PD-L1* showed significantly enhanced expression after recurrence (*p* < 0.005; *p* < 0.005). For *PD-L1* expression in recurrent gliomas, the adjuvant therapy group showed significantly increased expression compared to primary gliomas (*p* < 0.005). For *PD-1*- primary gliomas, if the matched recurrent gliomas showed *PD-1*+, the PFS became worse than the remaining recurrent gliomas *PD-1*- (12.7 vs. 25.9 months, *p* = 0.032). Interestingly, for *PD-L1*- primary gliomas, if the matched recurrent gliomas showed *PD-L1*+, the OS became better than the remaining recurrent gliomas *PD-L1*- (33.8 vs. 17.5 months, *p* < 0.001). **Conclusions:** In the study, we found the expression of *PD-1*/*PD-L1* increased significantly in recurrent gliomas and the elevated level of *PD-L1* was tightly associated with adjuvant treatment, suggesting the potential therapeutic and predictive value of *PD-1* and *PD-L1* in the treatment of recurrent gliomas.

## 1. Introduction 

Glioma accounts for 36.7–42.6% of all primary brain tumors, which is the most common primary brain neoplasm [1,2,3]. Gliomas can be histopathologically classified as a low-grade glioma or high-grade glioma. The median survival time for a low-grade glioma is 5–10 years. However, 50–75% of low-grade gliomas will eventually develop into a high-grade glioma. Glioblastoma is highly malignant and shows rapid clinical progression. Notably, glioblastoma accounts for the highest proportion of all gliomas [1,2]. The aggressive treatments on glioblastoma include surgical resection with a maximum safety margin, followed by concurrent chemoradiotherapy and six cycles of adjuvant temozolomide chemotherapy. Even after receiving all these treatments, the median survival time of patients with newly diagnosed glioblastoma is around 14.6 months [4,5,6]. Despite effective radiotherapy and chemotherapy after the operation, most patients will inevitably relapse after treatment. The recurrent glioma is more aggressive, progresses more rapidly, and resists treatment [7]. The treatment of recurrent gliomas has always been a challenge for clinicians. These years, the anti-tumor effects of immune checkpoint inhibitors have been increasingly recognized. They are highly reactive in bladder carcinoma, head and neck cancer, melanoma, lung cancer, and renal cell carcinoma with long-term tumor remission [8,9,10,11]. The brain is an organ with a unique tumor immune microenvironment [12]. The presence of tumor-infiltrating lymphocytes (TILs) in tumor lesions indicates that the immune modulation is involved in brain neoplasm progression [13]. Tumors generate immunosuppressive systems to avoid host immune attacks [14]. *Programmed death protein 1* (*PD-1*) and *programmed death-ligand 1* (*PD-L1*) play critical roles in tumor immune escape [15]. In 2000, Freeman et al. confirmed that *PD-1* could reverse modulate lymphocyte activation [16]. *PD-1*, a co-inhibitory receptor, is expressed in activated *CD4*+ or *CD8*+ T cells. *PD-L1*, the ligand of *PD-1*, is represented by a variety of cells, including tumor cells and lymphocytes. The reaction between *PD-1* and *PD-L1* in T cells could exhaust T cell clones, allowing tumor cells to survive under normal physiological conditions [17,18,19].

*PD-L1* expression in tumors, validated by immunohistochemistry (IHC) assays, is related to the response of *PD-L1/PD-1* inhibitors. Besides, it is suggested as a biomarker in clinical practice [9,20]. Multiple studies show the evidence that *PD-1/PD-L1* is expressed in gliomas [21,22,23]. However, up to now, few systematic studies have compared the expression of immune checkpoint molecule *PD-1/PD-L1* genes in primary and matched recurrent specimens of glioma.

Data comparing paired primary and recurrent gliomas are scarce, due to the small proportion of reoperation in recurrent gliomas. In this study, 42 patients who underwent surgical resections of both primary and recurrent gliomas were retrospectively included. *PD-1/PD-L1* expression in tumors was assessed by IHC. This comparative study will help us better understand the tumor biology of glioma, thus providing new insights into the treatment strategy.

## 2. Materials and Methods 

### 2.1. Patients

From January 2008 to December 2015, 42 patients who received surgical resections of both primary and recurrent gliomas in the Second Affiliated Hospital of Zhejiang University (SAHZU) were retrospectively included. Histopathology diagnosis was acquired after operations and diagnosed by neuropathologists. Patients were included if they met the following inclusion criteria: (1) patients received operations of both primary and recurrent gliomas; (2) primary and recurrent gliomas were histopathologically confirmed; (3) long-term follow-up in SAHZU after treatment. On the other hand, patients were excluded based on the following: (1) patients received immune therapy; (2) one of the operations was not conducted in SAHZU; (3) lack of pathological confirmation. *PD-1, PD-L1* and *IDH1* expression in tumors was assessed by IHC. This study was approved by the Ethics Committee of SAHZU, and was carried out in accordance with the Declaration of Helsinki. The code of ethics was 046.

### 2.2. Immunohistochemistry

The primary antibodies used in this study were *PD-1* (Abcam, Cambridge, UK), *PD-L1* (Cell Signaling Technology, Danvers, MA, USA) and *IDH1* (ZSGB-BIO, Beijing, China). Formalin-Fixed and Paraffin-Embedded (FFPE) blocks from the pathology department of SAHZU were cut into serial 4 um slices with a microtome. The paraffin sections were incubated at 60 °C in the incubator overnight. The sections were then deparaffinized in xylene and rehydrated through graded alcohols (100%, 95%, 75%). The sections for *PD-1, PD-L1, IDH1* testing were put in boiled SignalStain EDTA Unmasking Solution (pH 9) in the electric cooker for 10 min and heat-preserved for 10 min. Then the sections were blocked with 1% BSA (Bovine serum Albumin) and incubated at 4 °C overnight with primary antibody (the dilution rate of the primary antibody was as follows: *PD-1* (1:50), *PD-L1* (1:200), *IDH1* (ready to use). The day after, sections were incubated with HRP-conjugated secondary antibody at room temperature (the second antibody used for *PD-1* and *IDH1* was the Polink-1 HRP staining system (ZSGB-BIO), incubated for 15 min. The second antibody used for *PD-L1* was SignalStain Boost IHC Detection Reagent (Cell Signaling Technology), incubated for 30 min). After that, the sections were incubated with chromagen 3, 3′-diaminobenzidine (DAB) at room temperature (10 min for *PD-1*, 2 min for *PD-L1* and *IDH1*). The sections were counterstained with hematoxylin for 3 min and dehydrated through graded alcohols (75%, 95%, 100%) and mounted. 

### 2.3. Scoring System

*PD-1* was expressed in lymphocytes, so the immunohistochemical staining evaluation of the *PD-1* protein was unique: to find the area with the highest density of TILs in low magnification, counting *PD-1* expression cell at a 400× visual field (/HPF). Staining was scored as positive if the number of *PD-1*-positive TIL was one cell per high-power field [24,25]. Evaluation of *PD-L1* protein expression by IHC staining was based on the range and the intensity. Briefly, the proportion of immunopositive cells among the total number of tumor cells was subdivided into five categories, as follows: 0, <1%; 1, 1–25%; 2, 25–50%; 3, 50–75%; and 4, >75% positive cells. The immunointensity was subclassified into four groups: 0, negative; 1, weak; 2, moderate; and 3, strong immunointensity. IHC scores were generated by multiplication of these two parameters. The value of multiplication less than two was defined as *PD-L1*-negative. The value of multiplication equal to or more than two was defined as *PD-L1*-positive. Immunohistochemical staining evaluation of the IDH1 protein was based on the intensity of staining. Negative staining was defined as wild-type, and positive staining was defined as a mutation.

### 2.4. Statistical Analysis

A normality test (Kolmogorov–Smirnov test) and homogeneity of variance test (Levene test) were conducted in measurement data, such as age, among subgroups. In each test, *p* > 0.1 was considered for the Gaussian distribution and homogeneity of variance, respectively. A two-group t-test was conducted among subgroups to compare the measurement data if they had a Gaussian distribution and homogeneity of variance. The chi-square test and Fisher’s exact test was conducted among subgroups to compare enumeration data, such as sex, tumor location, WHO grade, residue, adjuvant therapy, and type of recurrence. *p* < 0.05 was considered significant. For the ranked data of paired primary and recurrent samples, the Wilcoxon rank-sum test was used to analyze the variation trend of each gene expression. Correlations between *PD-1* and *PD-L1* gene expression were tested by the Spearman rank correlation test. Overall survival (OS) is defined as the time from the first surgical treatment until the death of the patient. Progression-free survival (PFS) is defined as the time from the first operation of the glioma to the progression of the tumor. PFS and OS were estimated by the Kaplan–Meier method with a two-sided log-rank test. *p* < 0.05 was considered statistically significant. IBM SPSS Statistics 26.0 was used for statistical analyses.

## 3. Results

### 3.1. Patients’ Characteristics and Follow-Up

**Figure 1:**
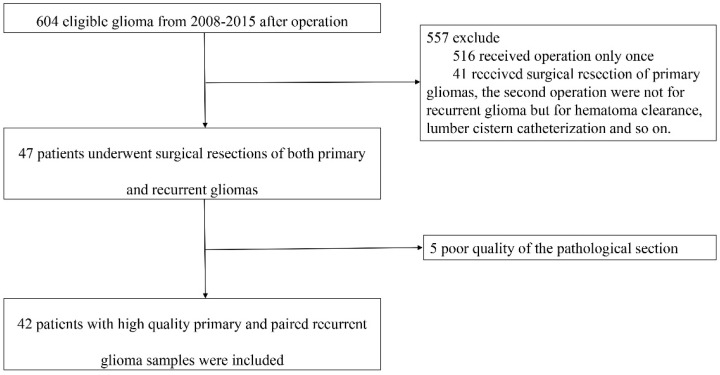
Study profile. Patients with high-quality primary and paired recurrent glioma samples were included.

From January 2008 to December 2015, 42 patients who received surgical resections of both primary and recurrent gliomas were retrospectively included (Figure 1). The genders of the patients were 27 male and 15 female. The average age at first operation was 43.2 years (range: 11–61 years old). After the first surgical resection, 21 cases had residual tumors, and 21 had no residual tumor. There were two pilocytic astrocytomas, eight astrocytomas with IDH1-mutant, 15 astrocytomas with IDH1-wildtype, three oligodendrogliomas, NOS, 12 glioblastomas with IDH1-wildtype, and one ganglioglioma. Nine patients were IDH1-mutant, 33 were IDH1-wildtype. Six patients received radiation alone, one patient received only chemotherapy, 15 patients received radiation and chemotherapy, and 20 patients had not received adjuvant therapy. Before the secondary operation, 38 cases recurred in situ, and four cases had a distant recurrence in the brain. After the second operation, 31 patients had residual tumors, and 11 patients had no residual tumor. There was one pilocytic astrocytoma, nine astrocytomas with IDH1-mutant, 10 astrocytomas with IDH1-wildtype, four oligodendrogliomas, NOS, and 18 glioblastomas with IDH1-wildtype. Eleven patients were IDH1-mutant, 31 were IDH1-wildtype. After that, one patient received only radiation, eight patients received chemotherapy alone, nine patients received radiotherapy and chemotherapy, and 24 patients had not received adjuvant treatment. The last follow-up time was 18 August 2017, with a median PFS of 13.5 months (95% CI: 11.0–16.05 months). The median PFS of newly diagnosed low-grade gliomas was 18 months (95% CI: 12.4–24.6 months), and the median PFS of the newly diagnosed high-grade gliomas was 12.7 months (95% CI: 10.0–15.4 months). At the time of the last follow-up, 12 patients were alive. The median OS was 33.8 months (95% CI: 28.3–39.3) for all 42 patients. The median OS of newly diagnosed low-grade gliomas was 54.9 months (95% CI: 30.9–78.9), and the median OS of the newly diagnosed high-grade gliomas was 30.0 months (95% CI: 22.2–37.8): 31.0 months (95% CI: 26.6–35.4) for grade III gliomas and 24.4 months (95% CI: 22.2–37.8 months) for glioblastomas. The median OS after the second operation was 14.1 months (95% CI: 7.7–20.5). In primary gliomas, 18 cases were low-grade gliomas, and 24 cases were high-grade gliomas. In recurrent gliomas, 34 cases were high-grade gliomas, and 8 cases were low-grade gliomas. Ten cases deteriorated from low-grade to a high-grade glioma (Table 1).

### 3.2. Increased Protein Expression of PD-1 and PD-L1 in Recurrent Gliomas Compared to Their Corresponding Primary Tumors

**Figure 2:**
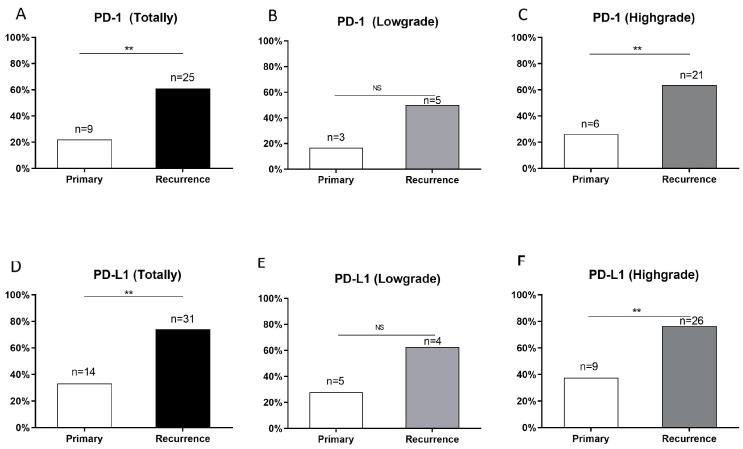
Expression of *PD-1* and *PD-L1* in primary and recurrent gliomas. (**A**–**C**) *PD-1* expression in primary and recurrent gliomas in the cohort of totality, low-grade and high-grade gliomas. (**D**–**F**) *PD-L1* expression in primary and recurrent gliomas in the cohort of totality, low-grade and high-grade gliomas. NS: no significance. **: *p* < 0.01.

In primary gliomas, expression of *PD-1* and *PD-L1* was found in 9 (22.0%) and 14 (33.3%) patients, respectively; in contrast, 25 (61.0%) and 31 (74.0%) of the lesions, respectively, had positive *PD-1* and *PD-L1* expression in their corresponding recurrent gliomas. In the samples from the first operation, *PD-1* and *PD-L1* expression was found in only 3 (16.7%) and 5 (27.8%), respectively, of patients with low-grade glioma; in high-grade glioma cases, the corresponding numbers of were 6 (26.1%) and 9 (37.5%), respectively. In the recurrent samples, low-grade gliomas expressed *PD-1* and *PD-L1* in 5 (62.5%) and 4 (50.0%), respectively, of the patients, whereas they were expressed in 21 (63.6%) and 26 (76.5%), respectively, of the patients with recurrent high-grade gliomas (Figure 2). For *PD-1* expression, 21 cases (51.2%) were shown to be negative in primary gliomas but positive in matched recurrent tumor samples; five cases (12.2%) were shown to be positive in primary gliomas but negative in matched recurrent tumor samples; 15 cases (36.6%) had no changes. The corresponding numbers for *PD-L1* were 24 (57.1%), 7 (16.7%), and 11 (26.2%), respectively. The overall expression rates of *PD-1* and *PD-L1* were enhanced after recurrence (*p* < 0.005; *p* < 0.005). 

Ten low-grade glioma patients progressed to high-grade glioma: For *PD-1* expression, one patient showed positive expression in both the primary and recurrent samples; another patient changed from positive to negative. In the rest of the eight patients with *PD-1*-negative expression in primary glioma, three of them (3/8, 37.5%) changed to positive when there was recurrence. For *PD-L1* expression, one patient showed positive expression in both the primary and recurrent samples; two patients changed from positive to negative. In the rest of the seven patients with *PD-L1*-negative expression in primary glioma, five of them (5/7, 71.4%) changed to positive when there was recurrence. 

**Figure 3:**
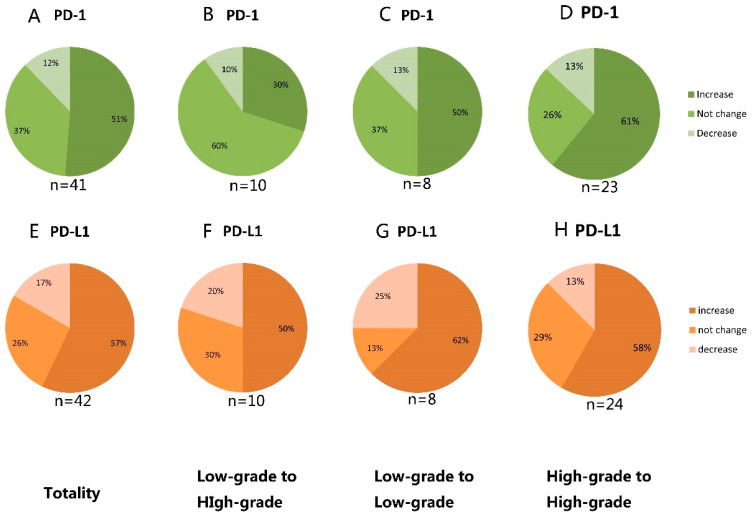
Change in *PD-1* (**A**–**D**) and *PD-L1* (**E**–**H**) expression from primary to recurrent glioma. (**A**) In the whole group, 51% of cases with *PD-1* expression increased from primary to recurrent glioma, 37% with *PD-1* expression were unchanged, and 12% with *PD-1* expression decreased. (**B**) Ten cases were low-grade gliomas at the first operation, and developed to high-grade when there was recurrence. Thirty percent of cases with *PD-1* expression increased from primary to recurrent glioma, 60% with *PD-1* expression were unchanged, and 13% with *PD-1* expression decreased. (**C**) Eight cases were low-grade gliomas at the first operation, and continued to be of low grade when there was recurrence. Fifty percent of cases with *PD-1* expression increased from primary to recurrent glioma, 37% with *PD-1* expression were unchanged, and 13% with *PD-1* expression decreased. (**D**) Twenty-three cases were high-grade gliomas at first operation, and were still high-grade gliomas when there was recurrence. Sixty-one percent of cases with *PD-1* expression increased from primary to recurrent glioma, 26% with *PD-1* expression were unchanged, and 13% with *PD-1* expression decreased. (**E**) In the whole group, 57% of cases with *PD-L1* expression increased from primary to recurrent glioma, 26% with *PD-L1* expression were unchanged, and 17% with *PD-L1* expression decreased. (**F**) Ten cases were low-grade gliomas at first operation and developed to high-grade when recurrence. Fifty percent of cases with *PD-L1* expression increased from primary to recurrent glioma, 30% with *PD-L1* expression were unchanged, and 20% with *PD-L1* expression decreased. (**G**) Eight cases were low-grade gliomas at the first operation and continued to be low-grade when there was recurrence. Sixty-two percent of cases with *PD-L1* expression increased from primary to recurrent glioma, 13% with *PD-L1* expression did not change, and 25% with *PD-L1* expression decreased. (**H**) Twenty-four cases were high-grade gliomas at first operation, and continued to be high-grade gliomas when there was recurrence. Fifty-eight percent of cases with *PD-L1* expression increased from primary to recurrent glioma, 29% with *PD-L1* expression were unchanged, and 13% with *PD-L1* expression decreased.

**Figure 4:**
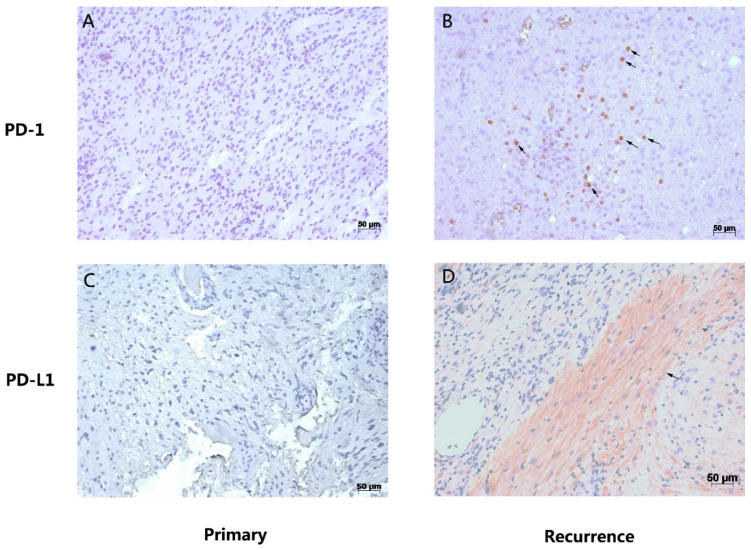
Expression of *PD-1* and *PD-L1* in two representative patients with primary and paired recurrent gliomas. One patient was diagnosed as having *PD-1*-negative glioblastoma after the first operation in 2013, and the histological diagnosis was still glioblastoma (**A**), with recurrence in 2014, but where the expression of *PD-1* was positive (**B**). Another patient was diagnosed as having *PD-L1*-negative anaplastic astrocytoma after the first operation in 2012 (**C**), and the histological diagnosis was glioblastoma when there was recurrence in 2014, and the expression of *PD-L1* was positive (**D**).

In cases which were high-grade gliomas at first operation, they are still high-grade when there is recurrence. For *PD-1* expression, three patients showed positive expression in both the primary and recurrent samples; three patients changed from positive to negative. In the rest of the 17 patients with *PD-1*-negative expression in primary glioma, 14 of them (14/17, 82.4%) changed to positive when there was recurrence. For *PD-L1* expression, six patients showed positive expression in both the primary and recurrent samples; three patients changed from positive to negative. In the rest of the 15 patients with *PD-1*-negative expression in primary glioma, 14 of them (14/15, 93.3%) changed to positive when there was recurrence (Figure 3). Figure 4 showed the expression of *PD-1* and *PD-L1* in two typical patients in both their primary and recurrent gliomas. 

### 3.3. Effect of Postoperative Adjuvant Therapy on the Expression of PD-1, PD-L1

To evaluate the impact of postoperative adjuvant treatment on the expression of *PD-1* and *PD-L1* genes, we further classified patients into the adjuvant therapy group (*n* = 22) and no adjuvant therapy group (*n* = 20). Compared with primary gliomas, the overall expression of *PD-1* increased in the no-adjuvant therapy group (*p* < 0.05), and it had an increasing trend in the adjuvant therapy group (0.05 < *p* < 0.1). Compared with primary gliomas, the overall expression of *PD-L1* increased in the adjuvant therapy group (*p* < 0.005), but the no adjuvant therapy group showed no statistical differences (*p* > 0.25) (Table 2).

### 3.4. Correlation between PD-1/PD-L1 Expression

**Figure 5:**
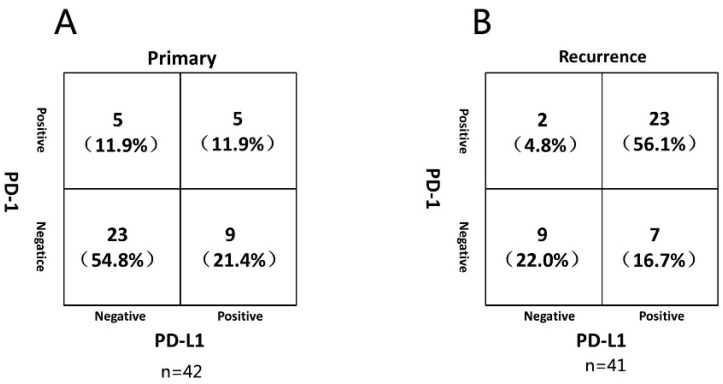
Correlation between *PD-1* and *PD-L1* in primary (**A**) and recurrent (**B**) gliomas.

**Figure 6:**
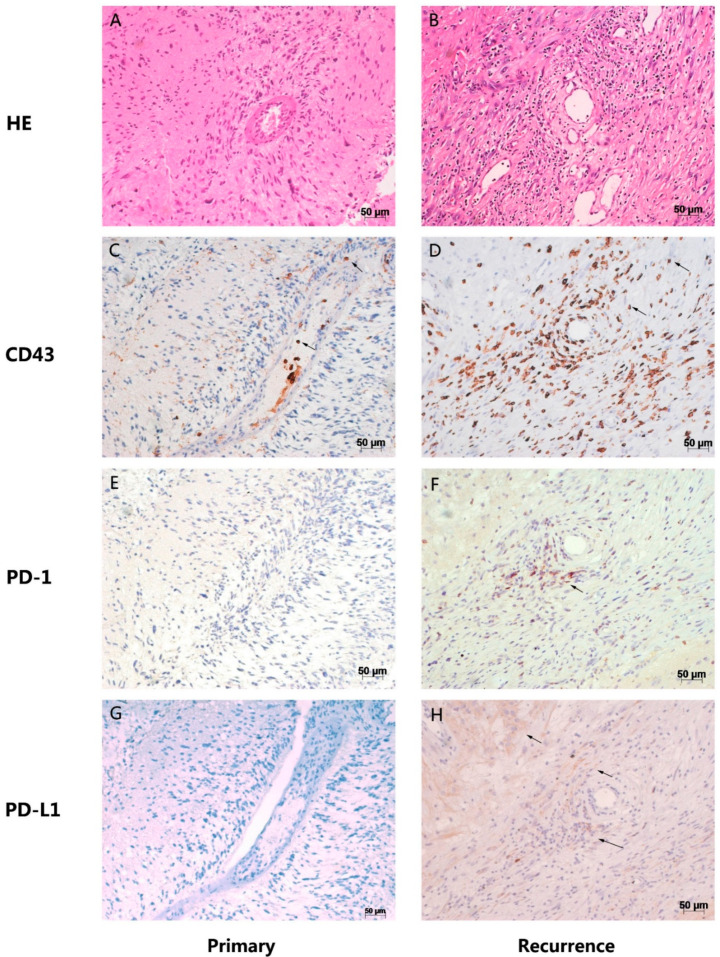
*PD-1* and *PD-L1* expression in the same location in a primary tumor and its corresponding recurrent glioma in one of the glioma patients. (**A**) HE staining for primary glioma. (**B**) HE stained for the corresponding recurrent glioma. (**C**) *CD43* immumohistochemical staining for the serial section of primary glioma. (**D**) *CD43* immumohistochemical staining for the serial section of the corresponding recurrent glioma. (**E**) *PD-1* immumohistochemical staining for the serial section of primary glioma. (**F**) *PD-1* immumohistochemical staining for the serial section of the corresponding recurrent glioma. (**G**) *PD-L1* immumohistochemical staining for the serial section of primary glioma. (**H**) *PD-L1* immumohistochemical staining for the serial section of the corresponding recurrent glioma. The patient was diagnosed as glioma grade III, negative for both *PD-1* and *PD-L1* after the first operation in 2011; when recurrence occurred, the histological diagnosis progressed to glioblastoma with positive *PD-1* and *PD-L1. PD-1* was expressed in lymphocytes (*CD43* labeled), and *PD-L1* was expressed in tumor cells near these lymphocytes.

Recent studies indicated that co-expression of *PD-1* and *PD-L1* might be correlated with responses to immune checkpoint inhibitors [23]. Thus, we investigate the correlation between *PD-1/PD-L1* expression in glioma samples. In primary samples, co-expression of *PD-1* and *PD-L1* was evident in 5 (11.9%) glioma samples and negative staining for both *PD-1* and *PD-L1* in 23 (54.8%) samples. *PD-1* expression was not correlated with *PD-L1* status (rs = 0.198, *p* = 0.21) (Figure 5A). Notably, in recurrent samples, co-expression of *PD-1* and *PD-L1* was found in 23 (56.1%) samples and negative staining for both *PD-1* and *PD-L1* in 9 (22.0%) samples. *PD-1* expression showed a significant positive correlation with *PD-L1* expression (rs = 0.531, *p* < 0.001) (Figure 5B). Figure 6 shows *PD-1* and *PD-L1* expression in the same location in a primary tumor and its corresponding recurrent glioma from one of the glioma patients. This typical case was diagnosed as glioma grade III after surgical resection of primary glioma in 2012 with both the *PD-1* and *PD-L1*-negative stains. Strikingly, when recurrent, the pathologic diagnosis was glioma grade IV after secondary surgery in 2014 with both *PD-1* and *PD-L1*-positive staining. Here, we used *CD43* as a lymphocyte marker to the located *PD-1*-positive cells. Results indicated that *PD-1* was expressed in tumor-infiltrating lymphocytes, whereas *PD-L1* was expressed in tumor cells near these lymphocytes (Figure 6). 

### 3.5. The Prognostic Value of PD-1 and PD-L1

**Figure 7:**
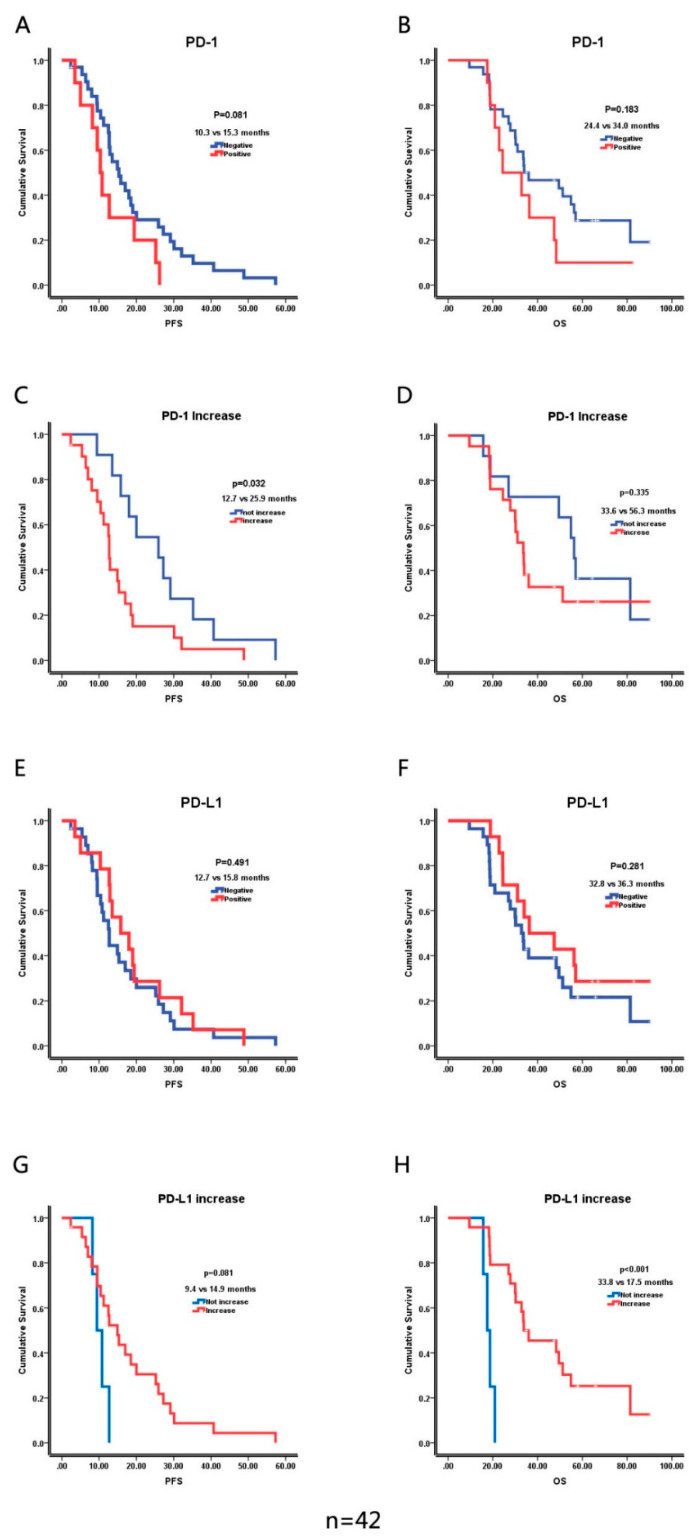
Correlation of the expression of *PD-1, PD-L1*, increasing *PD-1*, and increasing *PD-L1* with PFS and OS. (**A**) Comparation of progress-free survival (PFS) in *PD-1* negative group and *PD-1* positive group. (**B**) Comparation of overall survival (OS) in *PD-1* negative group and *PD-1* positive group. (**C**) Comparation of PFS in the group that *PD-1* expression didn’t increase after recurrence and *PD-1* expression increased after recurrence. (**D**) Comparation of OS in the group that *PD-1* expression didn’t increase after recurrence and *PD-1* expression increased after recurrence. (**E**) Comparation of PFS in *PD-L1* negative group and *PD-L1* positive group. (**F**) Comparation of OS in *PD-L1* negative group and *PD-L1* positive group. (**G**) Comparation of PFS in the group that *PD-L1* expression didn’t increase after recurrence and *PD-L1* expression increased after recurrence. (**H**) Comparation of OS in the group that *PD-L1* expression didn’t increase after recurrence and *PD-L1* expression increased after recurrence.

Table 3 compared baseline data of the *PD-1*-negative and *PD-1*-positive group. Table 4 showed baseline data of the *PD-L1*-negative and *PD-L1*-positive groups. Univariate analysis indicated that *PD-1* and *PD-L1* expression was not predictive of PFS or OS in primary and recurrent glioma. Importantly, for *PD-1*-negative primary gliomas, if the matched recurrent gliomas showed positive *PD-1* expression, the PFS became worse than the recurrent gliomas that remained *PD-1*-negative (12.7 vs. 25.9 months, *p* = 0.032). Interestingly, for *PD-L1*-negative primary gliomas, if the matched recurrent gliomas showed positive *PD-L1* expression, the OS became better than recurrent gliomas that remained *PD*-1-negative (33.8 vs. 17.5 months, *p* < 0.001) (Figure 7).

## 4. Discussion

**Figure 8:**
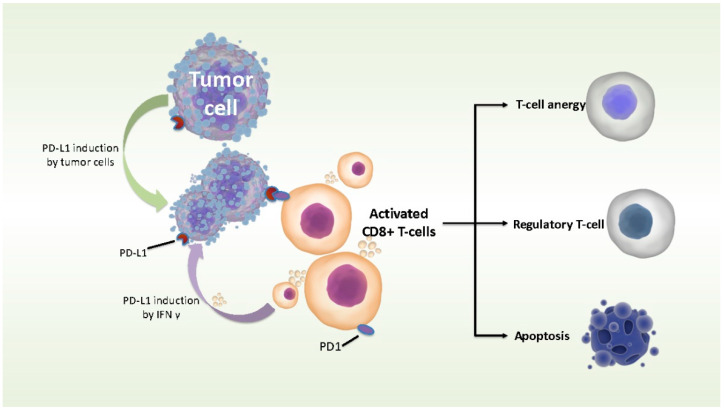
*PD-L1* is upregulated on cancer cells by IFN-γ released from infiltrating immune cells. Meanwhile, it is also upregulated by a tumor-specific non-IFN-γ-dependent mechanism. As a receptor of cancer cells, *PD-L1* induces a killing effect on cytotoxic T lymphocyte (CTL) by interacting with *PD-1* expressing tumor infiltrating lymphocyte (TIL) to inhibit the antitumor immune responses.

PD signaling represents a tumor adaptation in the context of cancer. Tumor cells can restrain immune responses by endogenous cellular feedback, which is called “adaptive resistance”. The combination of tumor PD-L1 and the PD-1 receptor on infiltrating effector T cells can inhibit cytotoxic activity. As a result, the CTL (cytotoxic T lymphocyte)-mediated elimination of cancer cells is inhibited (Figure 8). Elevated PD-L1 levels in tumor cells are connected to more aggressive behaviors and poor prognoses in certain cancers, including the breast, pancreas, kidney, ovary, gastric and esophageal cancer. To date, there is no systematic study on *PD-1/PD-L1* expression patterns in primary and paired recurrent glioma specimens. Berghoff et al. used immunohistochemistry to detect *PD-1/PD-L1* gene expression in 117 newly diagnosed glioblastomas and 18 recurrent glioblastomas. No significant difference in the expression of *PD-1* and diffuse/fibrillary *PD-L1* was found between the two groups. The expression of interspersed epithelioid tumor cells with membranous *PD-L1* in primary tumor cells was higher than that in recurrent specimens [26]. Rahman et al. analyzed 146 primary and 19 recurrent glioblastoma samples from TCGA datasets. They found no statistical difference in *PD-1* or *PD-L1* between primary and recurrent glioblastomas [27]. However, their studies have several limitations. The case number of recurrent gliomas in these studies is minimal, and they did not compare the paired primary and recurrent glioma, which cannot correctly answer the current question.

This study is the first to systematically compare the protein expression of the *PD-1/PD-L1* in primary and paired recurrent glioma tissue samples. Immunohistochemistry is currently the standard method to analyze the protein expression of *PD-1* and *PD-L1* in surgical specimens, which we used to detect the *PD-1/PD-L1* gene in 42 newly treated and paired recurrent glioma specimens. The expressions of *PD-1* and *PD-L1* in the recurrent samples were higher than those in the primary samples. As *PD-1* and *PD-L1* are immune escape-related genes, the increased expression of *PD-1* and *PD-L1* means that the tumor is more immune-tolerant. To investigate the effect of postoperative adjuvant therapy on the expression of *PD-1* and *PD-L1*, we further analyzed the cases in subgroups according to the situation of radiotherapy and chemotherapy. The results showed that in the chemoradiotherapy group, the expression of *PD-L1* in the recurrent specimens was significantly higher than that of the untreated samples, and *PD-1* had an increasing trend from primary to recurrence. The expression of *PD-1* was also significantly increased in the non-chemoradiotherapy group, but there was no significant difference in the expression of *PD-L1*. Our results suggest that the expression of *PD-1* in recurrent specimens is generally increased, which is not related to therapy, while the increase of *PD-L1* expression was more evident in patients who received adjuvant treatment. The expression rates of *PD-1* and *PD-L1* in primary glioma were 22% and 33%, respectively, and 61% and 74% in recurrent specimens. Berghoff et al. detected *PD-1/PD-L1* gene expression in 117 newly diagnosed glioblastomas and 18 recurrent glioblastomas. The expression rates of *PD-1* and *PD-L1* in untreated glioblastomas were 29.1% and 88%, respectively, and that of *PD-L1* in recurrent glioblastomas was 72.2%. Garber et al. analyzed *PD-1* protein expression in 235 glioma samples and *PD-L1* protein in 345 glioma samples and found that the expression rates of *PD-1* and *PD-L1* were 31.5% and 6.1%, respectively [24]. 

The expression rate of *PD-L1* was varied in different studies. Such variation might be caused by the different antibody used, the different scoring criteria, the heterogeneity of samples, and the different sample sizes. Berghoff et al. used the antibody made by Yale University Laboratory, the scoring standard was according to the staining range, and the specimen was glioblastoma [26]. Garber et al. used the antibody of Spring Biosciences and the scoring standard was according to the staining intensity, and the specimen was glioma [24]. However, we used the commercial antibody of cell signaling technology to identify glioma by staining range and staining intensity. We also found that *PD-1* and *PD-L1* expression were not correlated in primary glioma (Figure 4), somewhat at odds with the results reported by Garber et al. [24]. These findings may suggest that the *PD-1*/*PD-L1* pathway is activated to some extent in recurrent glioma, which means more severe immune suppression in recurrent glioma. Thus, the anti-*PD-1* and *PD-L1* antibodies used in glioma, especially in recurrent glioma, may be helpful. 

We further studied the correlation between *PD-1*, *PD-L1* gene expression, and survival, and found that the expression of *PD-1* and *PD-L1* was not correlated with PFS and OS. Berghoff et al. analyzed the expression of the *PD-L1* protein in 446 glioma samples from the TCGA database by univariate analysis and multivariate analysis, and found that the expression of *PD-L1* was not related to overall survival. Besides, Nduom et al. analyzed *PD-1* and *PD-L1* mRNA expression by univariate analysis and found that they were associated with poor prognosis [28]. Hao et al. conducted a meta-analysis and found *PD-L1* high expression predicted poor prognosis in glioblastoma [29]. However, we found that patients in which *PD-1* expression increased from primary to recurrent glioma relapsed more efficiently than those whose *PD-1* expression did not increase. More interestingly, an increase in *PD-L1* from primary glioma to recurrent glioma was a better predictor of prognosis than was a lack of increase. As we know, *PD-1* prevents the activation of T-cell cytotoxicity, with or without interaction with its ligand. Therefore, cytotoxic T-cell activation is further suppressed in recurrent glioma. We conjecture that in the group whose *PD-L1* did not increase, a more effective tumor-promotion mechanism than *PD-L1* occurred in their recurrent glioma. This finding may partly explain the explosive growth of gliomas after a stationary phase achieved using an immune checkpoint inhibitor. However, in our study, only four patients were *PD-L1*-negative in both their primary and recurrent gliomas; we need a larger sample size to corroborate this hypothesis. 

In recent years, immunosuppressive agents have shown considerable prospects in clinical practice. They are highly reactive in bladder cancer, head and neck cancer, melanoma, lung cancer, and renal cell carcinoma with persistent tumor remission [8,9]. The antibody targeting *PD-1* and *PD-L1* can reverse the immunosuppression, thus playing an anti-tumor effect [30]. An antibody directed to the *PD-1 / PD-L1* axis has shown a substantial impact on the mouse glioma model [31]. Currently, many clinical trials are underway on the use of these antibodies in gliomas—a phase III randomized controlled clinical trial combined nivolumab and ipilimumab in the treatment of glioblastoma (NCT02017717). A phase II clinical trial applied pembrolizumab alone or combined with bevacizumab to treat recurrent glioblastoma (NCT02337491). A phase II clinical trial combined atezolizumab with radiation and temozolomide during the concurrent stage and in combination with temozolomide during the adjuvant stage in newly diagnosed glioblastoma (NCT03174197), while Checkmate 143 reported that nivolumab, which targets *PD-1*, did not show survival benefits compared with bevacizumab in recurrent glioblastoma [32]. Possible etiologies of treatment failure may be systemic lymphopenia when recurrence, low *PD-L1* expression rate in this cohort, poor drug penetration of the blood–brain barrier (BBB), antigen-specific T-cells heavily dysfunctional in this special immune microenvironment, no identification of discrepancies between different genomic subtypes in their response to *PD-1/PD-L1* checkpoint blockades, or the presence of structural barriers preventing T-cell–antibody interactions. Therefore, two other studies followed. However, the Phase III CheckMate-498 study did not meet the primary endpoint of OS with nivolumab plus radiation in patients with newly diagnosed MGMT-unmethylated glioblastoma. CheckMate-548, which compared nivolumab or placebo with radiotherapy plus temozolomide (TMZ) in patients with newly diagnosed glioblastoma with a methylated *MGMT* promotor, obtained a negative result. Although these results are disappointing, many new methods to solve these potential causes of failure and new explorations are ongoing. A team from the Department of Immunobiology, Yale University School of Medicine found that the existence of GBM in the brain alone is not enough to cause an immune antitumor effect. The activation of a peripheral immune response may help to prolong the survival of patients with GBM. Their established VEGF-C-mRNA (lymphangiogenesis-promoting factor, Vascular Endothelial Growth Factor C (VEGF-C), using AAV9 and mRNA delivery vectors) can increase the activation of tumor-specific T cells and their tumor infiltration in lymph nodes. Combined administration of VEGF-C-mRNA and the anti-*PD-1* antibody resulted in tumor regression and survival benefits in tumor-bearing mice in a T-cell-dependent manner [33]. Hwang et al. reported that laser interstitial thermotherapy (LITT) can destroy BBB and may enhance host T-cell-mediated cytotoxicity. The combination of LITT and pembrolizumab in recurrent IDH wild-type glioblastoma prolonged PFS and OS [34,35]. A phase II clinical trial NCT03661723 adopted reirradiation-combined Pembrolizumab to stimulate the immune response and open up the blood–brain barrier, and achieved further stratification with Bevacizumab Naïve and Bevacizumab Resistant Recurrent Glioblastoma to accurately capture the group benefiting from *PD-1* antibody-combined irradiation [36]. There are also similar studies, such as NCT03743662 [37], NCT03197506 [38], NCT04977375 [39]. NCT03233152 adopted intra-tumoral ipilimumab plus intravenous nivolumab following the resection of recurrent glioblastoma for circumvention of BBB and stronger immune activation [40]. NCT04013672 used SurVaxM (a survivin vaccine) plus pembrolizumab for recurrent glioblastoma [41]. A single-arm phase II clinical trial (NCT02550249) concluded that neoadjuvant nivolumab modifies the tumor immune status in resectable glioblastoma [42]. A randomized, multi-institution clinical trial conducted by the Ivy Foundation Early-Phase Clinical Trials Consortium demonstrated that neoadjuvant anti-*PD-1* immunotherapy promotes a survival benefit with intratumoral and systemic immune responses compared to the adjuvant-only group in recurrent glioblastoma [43].

Current data are not mature and early studies have not shown clear benefits, yet we cannot rule out the possibility of using immune checkpoint inhibitors as a potential strategy for glioma. Our results indicated that the protein expression of *PD-1* and *PD-L1* increased in recurrent gliomas. Several clinical trials have demonstrated that the expression of *PD-1* and *PD-L1* is correlated with treatment response [9,44]. Immune checkpoint inhibitors targeting *PD-1* and *PD-L1* may have potential value in the treatment of recurrent gliomas.

## 5. Conclusions

This study explored the *PD-1/PD-L1* protein expression in recurrent glioma and its paired primary tumor. We confirmed the tendency of increased *PD-1/PD-L1* in recurrent glioma. In addition, increased *PD-L1* expression was related to adjuvant treatment. We revealed some immune characteristics of primary and their paired recurrent gliomas to a better understanding of gliomas, which indicated the potential therapeutic and predictive value of *PD-1* and *PD-L1* in the treatment and diagnosis of recurrent gliomas. Although several large-scale clinical trials obtained negative results of *PD-1* antibody applied in glioblastoma, further studies are needed to recognize the special immune microenvironment of the brain, especially under the tumor condition. We believe the dawn is just around the corner.

## Figures and Tables

**Table 1:** Baseline clinical characteristics.

Characteristics	Primary (*n* = 42)	Recurrence (*n* = 42)
**Age** **(year)**		
Mean (range)	43.2 (11–61)	44.9 (12–64)
**Sex**		
Male	27	27
Female	15	15
**Tumor location—side**		
Left	22	19
Right	20	22
Middle	0	1
**Pathological Type**		
Pilocytic astrocytoma	2	1
Astrocytoma, IDH1-mutant	8	9
Astrocytoma, IDH1-wildtype	15	10
Oligodendroglioma, NOS	3	4
Glioblastoma, IDH1-wildtype	12	18
Ganglioglioma	1	0
Pleomorphic xanthoastrocytoma	1	0
**IDH1 status**	9 (mutant)/33 (wildtype)	11 (mutant)/31 (wildtype)
**WHO grade**		
1	2	1
2	16	7
3	12	11
4	12	23
**Residual after first surgery**		
Yes	21	31
No	21	11
**Adjuvant therapy after first surgery**		
Radiotherapy alone	6	1
Chemotherapy alone	1	8
chemoradiotherapy	15	9
No adjuvant therapy	20	24
**Type of recurrence**		
Recurrence in situ		38
Distant relapse in the brain		4
**Median PFS (month)**	13.5	
**Median OS (month)**	33.8	14.1

**Table 2:** Effect of postoperative adjuvant therapy on the expression of *PD-1* and *PD-L1*.

	Group (*n*)	Primary (%)		Recurrence (%)		
		Positive	Negative	Positive	Negative	*p*
*PD-1*	No adjuvant therapy (20)	3	17	10	10	*p* < 0.05
	Adjuvant therapy (21)	6	15	15	6	0.05 < *p* < 0.1
*PD-L1*	No adjuvant therapy (20)	8	12	13	7	*p* > 0.25
	Adjuvant therapy (22)	6	16	18	4	*p* < 0.005

**Table 3:** Comparison of baseline clinical characteristics between *PD-1*-negative and *PD-1*-positive group in primary glioma.

Characteristics	*PD-1* Negative (*n* = 32)	*PD-1* Positive (*n* = 10)	*p*-Value
**Age** **(year)**			
Mean (range)	43 (11–61)	44 (12–61)	0.887
**Sex**			
Male	20	7	-
Female	12	3	1.000
**WHO grade**			
1	2	0	-
2	13	3	-
3	10	2	-
4	7	5	0.135
**IDH1 status**	9 (mutant)/23 (wildtype)	10 (wildtype)	0.086
**Resection type**			
Total gross resection	15	6	-
Subtotal resection	17	4	0.719
**Adjuvant therapy** **after first surgery**			
Yes	15	7	-
No	17	3	0.284
**Type of recurrence**			
Local recurrence	30	8	-
Distant recurrence	2	2	0.236
**Median PFS (month)**	15.3	10.3	0.081
**Median OS (month)**	34.0	24.4	0.183

**Table 4:** Comparison of baseline clinical characteristics between *PD-L1*-negative and *PD-L1*-positive groups in primary glioma.

Characteristics	*PD-L1* Negative (*n* = 28)	*PD-L1* Positive (*n* = 14)	*p*-Value
**Age** **(year)**			
Mean (range)	46 (11–61)	38 (12–52)	0.073
**Sex**			
Male	17	10	-
Female	11	4	0.734
**WHO grade**			
1	2	0	-
2	11	5	-
3	7	5	-
4	8	4	0.573
**IDH1 status**	7 (mutant)/21 (wildtype)	2 (mutant)/12 (wildtype)	0.692
**Resection type**			
Total gross resection	16	5	-
Subtotal resection	12	9	0.326
**Adjuvant therapy after first surgery**			
Yes	16	6	-
No	12	8	0.515
**Type of recurrence**			
Local recurrence	25	13	-
Distant recurrence	3	1	1.000
**Median PFS (month)**	12.7	15.8	0.491
**Median OS (month)**	32.8	36.3	0.284

## Data Availability

Data sharing not applicable to this article as no datasets were generated or analyzed during the current study.
